# Chronic Mercury Exposure and *GSTP1* Polymorphism in Munduruku Indigenous from Brazilian Amazon

**DOI:** 10.3390/toxics11020138

**Published:** 2023-01-31

**Authors:** Mayara Calixto da Silva, Rogério Adas Ayres de Oliveira, Ana Claudia Santiago de Vasconcellos, Bruno Hojo Rebouças, Bruna Duarte Pinto, Marcelo de Oliveira Lima, Iracina Maura de Jesus, Daniel Escorsim Machado, Sandra Souza Hacon, Paulo Cesar Basta, Jamila Alessandra Perini

**Affiliations:** 1 Research Laboratory of Pharmaceutical Sciences (LAPESF), State University of Rio de Janeiro (West Zone-UERJ-ZO), Rio de Janeiro 23070-200, Brazil; 2 Program of Post-Graduation in Public Health and Environment, National School of Public Health (ENSP), Oswald Cruz Foundation (Fiocruz), Rio de Janeiro 21040-900, Brazil; 3 Faculty of Medicine, University of São Paulo (USP), São Paulo 01246-903, Brazil; 4 Laboratory of Professional Education in Health Surveillance, Polytechnic School of Health Joaquim Venâcio (EPSJV), Fiocruz, Rio de Janeiro 21040-900, Brazil; 5 Environment Section (SEAMB), Evandro Chagas Institute (IEC), Health Surveillance Secretariat (SVS), Ministry of Health (MS), Ananindeua 67030-000, Brazil; 6 Department of Endemic Diseases Samuel Pessoa, ENSP, Fiocruz, Rio de Janeiro 21041-210, Brazil

**Keywords:** mercury exposure, *GSTP1*, genetic polymorphism, neurotoxicity, environmental health, indigenous people, Brazilian Amazon

## Abstract

Genetic polymorphisms may be involved with mercury levels and signs and symptoms of intoxication from this exposure. Therefore, the aims were to describe the frequency of the *GSTP1* polymorphism and to evaluate its effects on mercury levels and neurological signs in three Munduruku indigenous villages in the Brazilian Amazon. One-hundred-and-seven indigenous (over 12 years old) were included and genotyped (rs1695) using a TaqMan validated assay. Then, associations were evaluated by binary logistic regression, using odds ratios (OR) and 95% confidence intervals (CI). Mean age was 27.4 ± 13.9 years old, 52.3% were male, mean hair mercury concentration was 8.5 ± 4.3, exceeding the reference limit (≥6.0 µg/g), and were different among the three villages: 13.5 ± 4.6 µg/g in *Sawré Aboy*, 7.4 ± 2.3 µg/g in *Poxo Muybu* and 6.9 ± 3.5 µg/g in *Sawré Muybu*. The minor allele frequency of *GSTP1 G* was significantly different among the villages: 57% *Sawré Muybu,* 21% *Poxo Muybu* and 15% *Sawré Aboy.* Finally, after adjustment, *GSTP1 GG* and *GA* genotypes were associated with lower levels of Hg (OR = 0.13; CI95% = 0.03–0.49) and abnormal somatosensory signs (OR = 3.7; 95%IC = 1.5–9.3), respectively. In conclusion, monitoring this population is imperative to identify individuals at higher risk of developing signs of chronic mercury exposure based on the genetic profile.

## 1. Introduction

Mercury is the third most toxic element on the planet and can be found in different forms in the environment, of which organic mercury (methylmercury-MeHg) is the most dangerous chemical form and a global public health concern [1]. Artisanal small-scale gold mining (ASGM) is one of the main sources of human exposure to elemental mercury (Hg(0)), and approximately 3000 tons of Hg were released into the environment between 1975 and 2002, mostly in South America [2]. In the last years, it is estimated that ASGM (also called *garimpo*) increased over 90% in the Brazilian Amazon [3]. The Amazonian indigenous peoples need natural resources to live and the ASGM aftereffects threaten their livelihoods and cause health risks [4].

In water bodies, the elemental mercury undergoes methylation, mediated by aquatic micro-organisms, and forms MeHg, which bioaccumulates in fish. MeHg is able to overcome the blood-brain barrier and reach the central and peripheral nervous systems, causing changes in gait, coordination, cognition, visual dysfunctions and loss of skin sensitivity [5,6]. The indigenous peoples are one of the largest consumers of fish in the world [7] and consequently, they are at risk of higher concentrations of MeHg in their bodies [4,8].

Several studies showed an association between levels of mercury in the body and genetic polymorphisms, either conferring higher body levels of Hg or signs and symptoms of intoxication from this exposure [9,10,11,12] or providing protection against these effects [9,11,13]. Recently, the delta aminolevulinic acid dehydratase (*ALAD*) 177 *C>G* (rs1800435) polymorphism was identified in two indigenous children from the Brazilian Amazon, who showed elevated Hg levels (>9 µg/g), exceeding the reference limit (≥6.0 µg/g), and several neurological symptoms such as visual field alterations, memory deficit, distal neuropathy and toe amyotrophy [14]. Therefore, the study of polymorphisms in genes involved in MeHg metabolism is of great importance [4,14].

The MeHg metabolism involves its conjugation to small tripeptide glutathione (GSH), favoring the elimination via the ABC-transporter system in the bile [15]. The γ-glutamyl-cysteine ligase (GCL) enzyme synthesizes the GSH and then, the glutathione S-transferases, particularly the pi 1 isoform (GSTP1) catalyzes the conjugation of GSH to MeHg [16,17]. The GSTP1 enzyme is the most widely expressed glutathione-s-transferase (GST) in different parts of the human body, such as the liver, brain, lung, placenta, erythrocytes, muscle and others [18,19]. The GSTP1 enzyme is encoded by the *GSTP1* gene, a polymorphic member of the GST family, located on chromosome 11 (11q13.2) [20].

Single nucleotide polymorphisms (SNPs) in the *GSTP1* gene have been associated with MeHg retention and its symptoms of intoxication [13,20,21], with the rs1695 SNP standing out due to its elevated frequency in different populations and its ability to directly alter enzymatic activity due to the amino-acid substitution at position 105 from isoleucine to valine (Ile105Val), altering the geometry of the substrate binding site and reducing (approximately 3-fold) the substrate affinity in vitro [18,19,21,22].

Thus, the aims of this study were (1) to describe the frequency of the *GSTP1* rs1695 polymorphism in three Munduruku indigenous communities of the Brazilian Amazon and (2) to evaluate the effects of this SNP on the mercury levels and on the prevalence of neurological signs.

## 2. Materials and Methods

### 2.1. Study Design and Population

This study is part of a major project, described by Basta et al., 2021 [4] and approved by the National Ethics Committee of Human Research (protocol number 65671517.1.0000.5240). Here, a cross-sectional study was carried out with the Munduruku people of the middle Tapajós River, in Pará state, Brazilian Amazon, between October and November 2019, involving 107 indigenous adults from 3 villages (Sawré Muybu, Poxo Muybu and Sawré Aboy).

All residents over 12 years old were invited to participate in the study and there was no refusal. Therefore, a convenience sample was selected, without any probabilistic sampling methods. Then, all participants provided written informed consent and answered a questionnaire, which was applied by the research team during home visits and interviews with participating families [4].

### 2.2. Clinical and Neurological Evaluation

After the initial interview, participants were referred for clinical evaluation, where clinical and laboratory analysis were performed, and samples of hair and oral mucosa were collected for mercury exposure level determination and DNA extraction, respectively. The body mass index (BMI) was calculated as the weight (kg) divided by the square of height (m2). The Hemocue device was used to assess the hemoglobin (Hb) levels and determine the prevalence of anemia (Hb < 11.5 g/dL).

Participants underwent a systematized neurological examination protocol, specially developed for this research, carried out by three of the authors (RAAO, BDP and BHR). For somatosensory signs diagnosis, distal pinprick perception, distal thermal sensitivity, hallux or thumb vibration sensitivity and feet mechanical detection threshold were evaluated. Classical diagnostic research criteria for the diagnosis of peripheral polineuropathy, or neuropathy burden, were used [23] and included the presence of abnormalities in at least one somatic sensory domain and/or alterations in the ankle jerk reflex. Toe amyotrophy and ankle jerk reflex have been observed for the motor function diagnosis, while the brief cognitive screening battery (BCSB), verbal fluency test and stick design test were performed to evaluate the cognitive functions. The neurological assessment of the studied population is described in more detail elsewhere [6].

### 2.3. Hair Mercury Analysis

Mercury exposure levels were determined by the measurements of mercury in the hair of participants. Hair samples were removed with the aid of stainless-steel dissection scissors, close to the scalp in the occipital region and stored in paper envelopes, individually identified. Then, the samples were sent to the Toxicology Laboratory, in the Environment Section of the Evandro Chagas Institute (IEC), in Belém-Pará, Brazil, for an analysis of total mercury levels (THg). The complete applied methodology was described by Basta et al., 2021 [4].

Mercury exposure levels <6.0 µg/g in hair were considered as a safe health limit, following the WHO recommendations [24]. Therefore, levels of hair Hg were categorized in ≤6.0 µg/g or >6.0 µg/g, according to the safety dose recognized by WHO and the median observed in the studied population.

### 2.4. DNA Extraction and GSTP1 Genotyping

Samples from oral mucosa epithelial cells were collected using sterile swabs, stored in a buffered solution, individually identified, and transported to the Laboratory of Pharmaceutical Science-LAPESF of the State University of Rio de Janeiro, West Zone Campus, in Rio de Janeiro-RJ. The access to the genomic DNA was performed using an extraction kit (Qiagen, Hilden, Germany), following the procedures recommended by the manufacturer. Briefly, samples are incubated at 56 °C with 20 µL of proteinase and 400 µL of lysis buffer to release intracellular material. The mixture is then centrifuged and 400 µL of ethanol is added, allowing DNA precipitation. Then, 700 µL of the mixture is transferred to a silica column, which, after centrifugation, retains the DNA. After that, two washing steps are carried out to remove PCR inhibitors, such as divalent cations and proteins, followed by a full-speed spin to remove all traces of wash buffers from the silica column. Finally, we use a low-salt buffer to elute the purified, ready-to-use DNA.

The genotyping analysis of the *GSTP1* (chr11:67585218) A>G (rs1695) missense variant was performed using a TaqMan allelic discrimination assay (C_3237198_20) by a 7500 Real-Time System (Applied Biosystems, Foster City, CA, USA), as previously described [14] and the *GSTP1 A>G* allele frequency and genotype distribution were derived by gene counting.

### 2.5. Data Analysis

Continuous variables were presented as the mean, median, and their respective ranges. Linearity was tested with the Shapiro–Wilk normality test and the differences between groups for variables that did not present a normal distribution were evaluated by the Kruskal–Wallis (KW) nonparametric test. Categorical data were presented as number (n) and frequency (%) and analyzed using the chi-square test or Fisher’s exact test, if necessary. Deviations from Hardy–Weinberg equilibrium (HWE) in the *GSTP1 A>G* polymorphism frequency were assessed by the goodness-of-fit Chi-squared (χ^2^) test.

The associations between categorical variables and levels of mercury exposure were evaluated by determining the odds ratios (OR) and their respective 95% confidence intervals (95% CI), with adjustment for possible confounding factors, using binary logistic regression models. Age was considered a confounder for the association between the *GSTP1 A>G SNP* and Hg levels, while age and Hg levels were considered confounders for the association between the *GSTP1 A>G SNP* and the prevalence of neurological signs. All analyses were performed using IBM SPSS 20.0 Statistics for Windows (SPSS Inc., Chicago, IL, USA) and a significance level of 0.05 was adopted.

## 3. Results

The present study was conducted with 107 individuals older than 12 years old, residents from *Poxo Muybu* (31.8%), *Sawré Aboy* (21.5%) and *Sawré Muybu* (46.7%) villages. The majority of the individuals were male in *Poxo Muybu* and *Sawré Aboy* villages. The mean age in the overall population was 27.4 ± 13.9 years old (median 24.0, ranging from 12.0 to 72.0), with older individuals living in *Sawré Muybu* (58%) and younger individuals living in *Sawré Aboy* (60.8%). Approximately 5% of individuals showed anemia, and it was more common in residents of *Sawré Aboy*.

The mean level of mercury exposure in the studied population was 8.5 ± 4.3 (median 7.4, ranging from 2.0 to 22.8) and was not normally distributed (Shapiro–Wilk test *p*-value < 0.001). The *Sawré Aboy* village presented higher levels of mercury exposure when considering either the WHO cutoff point (6.0 µg/g) and the cutoff determined by the overall median of the studied population (7.4 µg/g). The *Sawré Aboy* residents also presented higher prevalence of abnormal somatosensory signs and cognitive functions (Table 1). When considering the prevalence of at least one abnormal neurological sign, 57.9% of individuals were affected in the overall population and a higher prevalence (82.6%) was found in *Sawré Aboy* residents (*p*-value = 0.004), comparing the three villages (data not shown).

**Figure 1:**
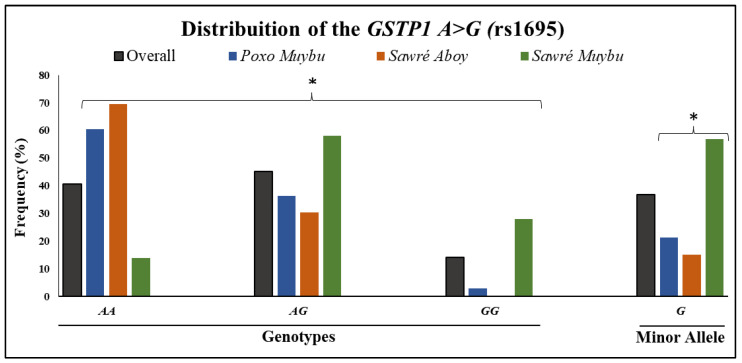
Distribution of the *GSTP1 A>G* (rs1695) genotypes (*AA*, *AG*, *GG*) and minor allele frequency (*G*) in the overall study population and in the 3 villages. * *p*-value < 0.0001 obtained from the χ^2^ test (Pearson *p*-value) when comparing the three villages, not including the overall group.

The rate of successful genotyping of the *GSTP1 A>G* SNP was 99.1%, since only a sample from one individual did not amplify during the PCR experiment. The distribution of the polymorphism was in Hardy–Weinberg equilibrium for the overall population (*p*-value = 0.79) and for each of the villages *Poxo Muybu, Sawré Aboy* and *Sawré Muybu* (*p*-values = 0.61, 0.39 and 0.19, respectively). The frequency distribution of the *GSTP1 A>G* polymorphism is presented in Figure 1. The distributions of the genotypes and minor allele frequency (MAF) among the three villages were statistically different (*p*-value < 0.0001), with *Sawré Muybu* residents presenting the highest frequency of the *GSTP1 GG* variant genotype and no individual from *Sawré Aboy* having that genotype. In addition, the *Sawré Aboy* village presented the highest frequency of the *GSTP1 AA* wild-type genotype.

To investigate the associations between the *GSTP1 A>G SNP* and Hg exposure (Table 2), we first considered the Hg levels categorized according to the WHO guidelines (≤6.0 µg/g or >6.0 µg/g). It was observed that the *GSTP1 GG* genotype was associated with lower levels of Hg exposure in both crude and adjusted analyses. Furthermore, this association is slightly stronger in the recessive model (*AA + AG* versus *GG*). Interestingly, the *Sawré Aboy* residents showed significant (*p*-value < 0.0001) higher levels of Hg (13.5 ± 4.6 µg/g; median = 12.5, range = 4.8–22.8) compared with *Poxo Muybu* (7.4 ± 2.3 µg/g; median = 7.20, range = 2.8–12.9; *p*-value < 0.0001) and *Sawré Muybu* (6.9 ± 3.5 µg/g; median = 6.38, range = 2.0–16.0, *p*-value < 0.0001), and no individual from *Sawré Aboy* presented the *GSTP1 GG* variant genotype (Figure 1), which was associated with protection (lower levels of Hg).

In addition, we investigated this association by considering the Hg levels categorized by the overall median (7.4 µg/g) and the results remained the same. Therefore, for the subsequent analysis, we considered only the Hg ≤ 6.0 (µg/g)^2^ versus > 6.0 (µg/g)^2^ classification.

Finally, the association between the *GSTP1 A>G* polymorphism and the prevalence of neurological signs was investigated. When considering the presence of any abnormal somatosensory sign, the *GSTP1 AG* genotype showed a positive association in both crude and adjusted analyses for age and Hg exposure levels (OR = 3.70 and 95% IC = 1.47–9.29; OR = 3.70 and 95% IC = 1.47–9.29, respectively), while the presence of abnormal motor or cognitive signs was not statistically significant (data not shown). In addition, each neurological sign individually and *GSTP1 A>G* polymorphism also investigated (Table 3 and Table 4). For the somatosensory signs, individuals carrying the *GSTP1 AG* genotype had an approximately 4-fold higher chance of having clinical signs of polyneuropathy, while the other signs were not significant among different *GSTP1* genotypes (Table 3). There was a low prevalence of abnormal motor functions in the studied population (4.7% for toe amyotrophy and 16.8% for abnormal ankle jerk reflex) and all individuals who presented toe amyotrophy were carriers of the *GSTP1 AG* genotype (*p*-value = 0.04), however, it was not possible to estimate the OR and the respective 95% IC (Table 4). Regarding the cognitive evaluation, no association was observed with *GSTP1* genotypes, however, the BCSB was significantly different among the groups, and approximately 42% of the altered results in this battery were from individuals carrying the *GSTP1 AA* genotype (Table 4), which was positively associated with higher levels of Hg in Table 2.

## 4. Discussion

The present study described the association of a single nucleotide polymorphism from a gene involved in the Hg metabolism and the presence of neurological signs of chronic mercury exposure in 107 indigenous adults residing in a region of the Brazilian Amazon affected by illegal mining activities. The Hg levels and *GSTP1 A>G* polymorphism genotype frequencies were significantly different among the three villages (*Poxo Muybu*, *Sawré Aboy* and *Sawré Muybu*). The *GSTP1 AA* genotype was associated with higher levels of Hg and the heterozygotic genotype (*GSTP1 GA*) was associated with a higher chance of having an abnormal somatosensory sign and neuropathy burden. In addition, the *Sawré Aboy* residents showed: (i) higher levels of Hg and (ii) higher frequency of the *GSTP1 AA* genotype.

Our findings retake the discussion initiated by Basta et al., 2021, who observed in this same population that Hg exposure levels and impaired neurological functions were higher in the *Sawré Aboy* village, which is located downstream from the Jamanxin River and is the closest village to the mining activities in comparison to the *Sawré Muybu* and *Poxo Muybu* ones [4,6]. In addition, these findings can also be explained by the higher frequency of the *GSTP1 AA* genotype, associated with higher Hg levels, in *Sawré Aboy* residents compared to the other two villages (*Sawré Muybu* and *Poxo Muybu*).

The distribution profile of the *GSTP1 A>G* (rs1695) polymorphism varies according to the population, with the MAF (allele *G*) ranging from 24.7% to 43% in the Brazilian population [25,26,27,28,29,30,31]. As far as we know, there is no data on *GSTP1* polymorphism frequency from other indigenous Brazilian populations focusing on mercury exposure. A recent study investigated the *GSTP1* frequency in the nonindigenous Brazilian population and observed that the MAF (ranging from 30.7% to 31.5%) did not differ between the groups based on skin color: black, mulatto, nonwhite and white [32]. Here, the *GSTP1* MAF in the overall indigenous population was 36.8%, similar (41.8%) to that described in the indigenous from the Amazon admitted to the Indian House of Health in Boa Vista, Roraima, Brazil (CASAI/RR), aiming to evaluate the genetic profile (*TP53* and *GSTP1* genes) and prostatic features [26]. However, a significant difference was found among the three villages: 21.2% *Poxo Muybu*, 15.2% *Sawré Aboy* and 57.0% *Sawré Muybu*. The *Munduruku* are an indigenous community from the Brazilian Amazon that lives isolated from other Amerindian populations and rarely display admixture with nonindigenous. Despite living near each other for centuries, these populations maintain their distinctiveness, according to the substantial differences found in their genetic profiles [4]. For this reason, it is not possible to extrapolate these results, not even for the same ethnic group. Therefore, the implementation of an individual genetic diagnosis is necessary.

The GST superfamily is comprised of 16 genes, including GSTP1, and encodes vital defense enzymes involved in the detoxification of reactive oxygen species and metal biotransformation [33,34]. GSTP1 is a key enzyme because it is the most widely expressed GST in the body. In addition to being frequent, the *GSTP1 A>G* non-synonymous polymorphism changes significantly the enzyme-substrate affinity [18,19,35,36,37]. The literature is not conclusive about the relationship between the *GSTP1 A>G* polymorphism and levels of Hg exposure. Our results contrast with studies where the minor allele *GSTP1 G* was associated with higher Hg levels from different exposure sources and biological matrices such as hair [9,38] and urine [13], and is agreement with other studies where the *GSTP1 G* showed a protective effect against elevated Hg levels dosed from hair [22] and blood samples [21,39]. One possible reason for the discrepancy among the studies is the use of different biological matrices to measure the Hg exposure, depending on the route of exposure, which may not present the same association with the polymorphism in the *GSTP1* gene, which is mostly involved with the metabolism of MeHg ingested from the diet [9,15,39]. Furthermore, this raises the question of whether Hg dosed from hair samples reflects the circulating levels of the metal or, instead, represents how much is being excreted from the organism. Nevertheless, the measurement of Hg in hair samples is advantageous due to the simple collection and transportation, which are easier than for blood and urine samples [40,41].

There is evidence that the *GSTP1* polymorphism is associated with several neurological conditions, such as multiple sclerosis [42] and autism spectrum disorders [43]. To date, however, no other clinical study has found an association between the *GSTP1 A>G* polymorphism and signs of neurotoxicity induced by MeHg. We believe that the enzymatic alteration caused by this SNP may alter the MeHg exposure levels in the peripheral and central nervous systems. This, in addition to the enhancement of oxidative stress, may play a role in the pathogenesis of the neurological abnormalities found in this series [44]. In the present study, despite the association found for the *GSTP1 AG* genotype, it is not possible to conclude that the SNP is involved with the onset of neurological signs and symptoms due to the small number of individuals enrolled and the low prevalence of these outcomes in the population. Furthermore, this might be due to the low prevalence of the neurological signs and symptoms of chronic mercury exposure and the low frequency of the *GSTP1 AA* genotype in *Sawré Muybu* and the *GSTP1 GG* genotype in *Poxo Muybu* and *Sawré Aboy*, ensuring a higher frequency of the *GSTP1 AG* genotype in the overall population. In addition, the individuals were young, and this kind of outcome is more prevalent in later ages, which could be better investigated in a longitudinal observation of this population, that also has an elevated frequency of polymorphism associated with mercury exposure levels.

Studies with indigenous communities have multiple ethical aspects that need to be considered, such as the cultural importance of the indigenous body and, therefore, all specimens that can be collected, such as tissue, blood, and hair [45]. In the present study, we were able to count on the engagement of community leaders, who made it possible to collect hair and oral mucosa samples and perform the needed analysis. This access was allowed thanks to the effort and competence of the multidisciplinary team that was assembled to evaluate this population, involving biologists, physicians, nurses, psychologists, and pharmaceutical geneticists. Efforts are being made to create a closer interaction between researchers and indigenous communities, to increase research recruitment and also to provide reports of individual results obtained with the studies [4]. This approach is especially important when genetic studies are carried out because individuals must be aware of the benefits of engaging in the research [4,14,45].

Although some limitations need to be discussed, such as nonmeasured variables that would be important to evaluate, such as occupation, alcohol consumption and smoking status, those can be sources of bias. Furthermore, the small sample size may lead to relatively weak power to detect the real association between the polymorphism and the neurological signs and symptoms. However, the potential benefits that can be provided to the communities must be considered. Therefore, in order to reduce some of these limitations and expand the clinical evaluation carried out among the *Munduruku* indigenous people, a longitudinal study is being designed to evaluate levels of mercury exposure in different matrices and the chronic effects of mercury exposure, in addition to identifying possible polymorphisms as susceptibility biomarkers of these events. Furthermore, the villages are in a high-risk area for ASGM since levels of Hg are extremely high, which reveals the extreme vulnerability of this population and the need for constant environmental and individual monitoring through a wide genetic evaluation that can easily be carried out with noninvasive biological matrices, such as the oral mucosa cells used in this work.

## 5. Conclusions

This is the first study to evaluate the association of the *GSTP1 A>G* polymorphism with Hg levels and the neurological signs that can be caused by chronic exposure to heavy metals in an extremely vulnerable population from the Brazilian Amazon. The Hg levels were significantly higher in *Sawré Aboy* residents, followed by those from *Poxo Muybu* and *Sawré Muybu*, while the *GSTP1 G* allele, which is associated with lower levels of Hg, was more frequent in *Sawré Aboy* residents, followed by *Poxo Muybu* residents, and finally *Sawré Muybu* residents. The *GSTP1 AA* and *GA* genotypes were associated with higher levels of Hg and a higher chance of having an abnormal somatosensory sign, respectively. Our findings highlight the importance of monitoring the indigenous communities in the Brazilian Amazon, who live in a vulnerable situation caused by the abandonment of the state. In conclusion, it is evident that further wide evaluation is imperative to this population, in order to identify individuals at higher risk of developing signs and symptoms of chronic mercury exposure. Thus, individual genetic diagnosis tests are crucial, since it is not possible to extrapolate data from other populations, even from other Amerindians.

## Figures and Tables

**Table 1:** Sociodemographic and clinical characteristics of the study population, according to the village of residence in *Sawré Muybu* indigenous territory, Pará State, Brazilian Amazon, 2019.

Characteristics	Overall (n = 107)	Village			
		*Poxo Muybu*	*Sawré Aboy*	*Sawré Muybu*	*p*-Value ^1^
		(n = 34)	(n = 23)	(n = 50)	
**Sex**					
Female	51 (47.7)	16 (47.1)	10 (43.5)	25 (50.0)	0.87
Male	56 (52.3)	18 (52.9)	13 (56.5)	25 (50.0)	
**Age (years)**					
12–19	36 (33.6)	15 (44.1)	11 (47.8)	10 (20.0)	**0.05**
20–24	19 (17.8)	5 (14.7)	3 (13.0)	11 (22.0)	
25–29	17 (15.9)	2 (5.9)	2 (8.7)	13 (26.0)	
≥30	35 (32.7)	12 (35.3)	7 (30.4)	16 (32.0)	
**BMI (kg/m^2^)**					
<18.5	3 (2.8)	2 (5.9)	1 (4.3)	0 (0.0)	0.54
18.5–24.9	66 (61.7)	19 (55.9)	16 (69.6)	31 (62.0)	
25.0–29.9	34 (31.8)	11 (32.4)	5 (21.7)	19 (36.0)	
≥30.0	4 (3.7)	2 (5.9)	1 (4.3)	1 (2.0)	
**Hemoglobin ^2^**					
≤11.5	5 (4.7)	1 (3.0)	3 (13.0)	1 (2.0)	0.1
>11.5	101 (95.3)	32 (97.0)	20 (87.0)	49 (98.0)	
**Hg levels (** **µ** **g/g) ^3^**					
≤ 6.0	32 (30.5)	10 (29.4)	1 (4.3)	21 (43.8)	**0.003**
> 6.0	73 (68.2)	24 (70.6)	22 (95.7)	27 (56.2)	
≤7.40	53 (50.5)	19 (55.9)	3 (13.0)	31 (64.6)	**<0.001**
>7.40	52 (49.5)	15 (44.1)	20 (87.0)	17 (35.4)	
**Somatosensory signs**					
Normal	66 (61.7)	30 (88.2)	10 (43.5)	26 (52.0)	**<0.001**
Abnormal	41 (38.3)	4 (11.8)	13 (56.5)	24 (48.0)	
**Motor functions**					
Normal	85 (79.4)	30 (88.2)	17 (73.9)	38 (76.0)	0.3
Abnormal	22 (20.6)	4 (11.8)	6 (26.1)	12 (20.4)	
**Cognitive evaluations**					
Normal	68 (63.6)	23 (67.6)	9 (39.1)	36 (72.0)	**0.02**
Abnormal	39 (36.4)	11 (32.4)	14 (60.9)	14 (28.0)	

^1^ *p*-value obtained from the χ^2^ test (Pearson *p*-value) or Fisher’s exact test, when needed. ^2^ Missing information from 1 individual. ^3^ Missing information from 2 individuals.

**Table 2:** Distribution of mercury exposure levels in the overall study population according to the *GSTP1 A>G* (rs1695) genotypes and alleles (*Sawré Muybu* indigenous territory, Pará State, Brazilian Amazon, 2019).

*GSTP1* (rs1695) *A>G*	Hg Levels (µg/g)				
	≤6.0	>6.0	*p*-Value ^1^	Crude OR (95% IC)	Adjusted OR ^2^ (95% IC)
	(n = 32)	(n = 73)			
**Genotypes**					
*AA*	11 (34.4)	32 (44.4)	**0.001**	1	1
*AG*	10 (31.2)	36 (50.0)		1.24 (0.47–3.30)	1.23 (0.46–3.30)
*GG*	11 (34.4)	4 (5.6)		**0.13 (0.03–0.48)**	**0.13 (0.03–0.49)**
					
*AA + GG*	21 (65.6)	68 (94.4)	**<0.001**	1	1
*GG*	11 (34.4)	4 (5.6)		0.11 (0.03–0.39)	0.12 (0.03–0.41)
					
**Alleles**					
*A*	32 (50.0)	100 (69.4)	**0.007**	1	1
*G*	32 (50.0)	44 (30.6)		**0.44 (0.24–0.81)**	**0.44 (0.24–0.81)**
***GSTP1* (rs1695) *A>G***	**Hg Levels (µg/g)**				
	**≤7.40**	**>7.40**	***p*-value ^1^**	**Crude OR** **(95% IC)**	**Adjusted OR ^2^ (95% IC)**
	**(n = 53)**	**(n = 52)**			
**Genotypes**					
*AA*	21 (39.6)	22 (43.1)	**0.05**	1	1
*AG*	20 (37.7)	26 (51.0)		1.24 (0.54–2.86)	1.24 (0.54–2.86)
*GG*	12 (22.6)	3 (5.9)		**0.24 (0.06–0.97)**	**0.24 (0.06–0.99)**
					
*AA + GG*	41 (77.4)	48 (94.1)	**0.02**	1	1
*GG*	12 (22.6)	3 (5.9)		**0.21 (0.06–0.81)**	**0.22 (0.06–0.83)**
					
**Alleles**					
*A*	62 (58.5)	70 (68.6)	0.13	1	1
*G*	44 (41.5)	32 (31.4)		0.64 (0.37–1.14)	0.65 (0.37–1.14)

n = 105, missing information genotype for 1 individual and Hg levels from 2 individuals. ^1^ *p*-value obtained from the χ^2^ test (Pearson *p*-value) or Fisher’s exact test, when needed. ^2^ Odds ratio adjusted for age.

**Table 3:** Distribution of the *GSTP1 A>G* (rs1695) genotypes and alleles in the overall study population according to the prevalence of somatosensory signs (*Sawré Muybu* indigenous territory, Pará State, Brazilian Amazon, 2019).

	GSTP1 (rs1695) *A>G* ^1^			
Somatosensory Signs	*AA*	*AG*	*GG*	*p*-Value ^2^
**Distal pinprick perception**				
Normal	37 (45.7)	33 (40.7)	11 (13.6)	0.15
Abnormal	6 (24.0)	15 (60.0)	4 (16.0)	
OR_c_ (95% IC)	1	2.80 (0.97–8.06)	2.24 (0.54–9.40)	
OR_a_ ^3^ (95% IC)	1	3.16 (1.07–9.31)	1.81 (0.39–8.43)	
				
**Distal thermal sensitivity**				
Normal	37 (42.0)	38 (43.2)	13 (14.8)	0.63
Abnormal	6 (33.3)	10 (55.6)	2 (11.1)	
OR_c_ (95% IC)	1	1.62 (0.54–4.92)	0.95 (0.17–5.30)	
OR_a_ ^3^ (95% IC)	1	1.79 (0.58–5.53)	0.67 (0.11–4.12)	
				
**Hallux or thumb vibration sensitivity**				
Normal	39 (41.1)	44 (46.3)	12 (12.6)	0.41
Abnormal	4 (36.4)	4 (36.4)	3 (27.3)	
OR_c_ (95% IC)	1	0.89 (0.21–3.78)	2.44 (0.48–12.45)	
OR_a_^3^ (95% IC)	1	0.98 (0.22–4.37)	1.84 (0.29–11.84)	
				
**Feet mechanical detection threshold**				
Normal	39 (42.9)	39 (42.9)	13 (14.3)	0.43
Abnormal	4 (26.7)	9 (60.0)	2 (13.3)	
OR_c_ (95% IC)	1	2.25 (0.64–7.92)	1.50 (0.25–9.16)	
OR_a_ ^3^ (95% IC)	1	2.50 (0.70–9.00)	1.05 (0.15–7.18)	
				
**Clinical signs of polyneuropathy** **^4^**				
No	34 (48.6)	26 (37.1)	10 (14.3)	**0.04**
Yes	9 (25.0)	22 (61.1)	5 (13.9)	
OR_c_ (95% IC)	1	3.20 (1.26–8.09)	1.89 (0.51–6.94)	
OR_a_ ^3^ (95% IC)	1	**3.67 (1.42–9.53)**	1.41 (0.35–5.68)	

OR_c_ = crude odds ratio. ^1^ Missing information from 1 individual. ^2^ *p*-value obtained from the χ^2^ test (Pearson *p*-value) or Fisher’s exact test, when needed. ^3^ Odds ratio adjusted (OR_a_) for age and Hg exposure levels. ^4^ Neuropathy burden.

**Table 4:** Distribution of the *GSTP1 A>G* (rs1695) genotypes and alleles in the overall study population according to the prevalence of motor and cognitive signs (*Sawré Muybu* indigenous territory, Pará State, Brazilian Amazon, 2019).

	*GSTP1* (rs1695) *A>G* ^1^			
Functions	*AA*	*AG*	*GG*	*p*-Value ^2^
**Motor**				
**Toe amyotrophy**				
Normal	43 (42.6)	43 (42.6)	15 (14.9)	**0.04**
Abnormal	0 (0.0)	5 (100.0)	0 (0.0)	
OR_c_ (95% IC)	1	-	-	
OR_a_ ^3^ (95% IC)	1	-	-	
				
**Ankle jerk reflex**				
Normal	36 (40.9)	40 (45.5)	12 (13.6)	0.94
Abnormal	7 (38.9)	8 (44.4)	3 (16.7)	
OR_c_ (95% IC)	1	1.03 (0.34–3.12)	1.29 (0.29–5.77)	
OR_a_ ^3^ (95% IC)	1	1.13 (0.36–3.52)	0.97 (0.19–4.99)	
				
**Cognitive**				
**BCSB ^4^**				
Normal	33 (40.2)	41 (50.0)	8 (9.8)	**0.04**
Abnormal	10 (41.7)	7 (29.2)	7 (29.2)	
OR_c_ (95% IC)	1	0.56 (0.19–1.64)	2.89 (0.84–9.95)	
OR_a_ ^3^ (95% IC)	1	0.58 (0.20–1.73)	3.51 (0.86–14.31)	
				
**Verbal fluency test**				
Normal	29 (38.7)	35 (46.7)	11 (14.7)	0.83
Abnormal	14 (45.2)	13 (41.9)	4 (12.9)	
OR_c_ (95% IC)	1	0.77 (0.31–1.89)	0.75 (0.20–2.79)	
OR_a_ ^3^ (95% IC)	1	0.79 (0.32–1.98)	1.05 (0.25–4.34)	
				
**Stick design test**				
Normal	42 (40.4)	47 (45.2)	15 (14.4)	0.84
Abnormal	1 (50.0)	1 (50.0)	0 (0.0)	
OR_c_ (95% IC)	1	0.89 (0.05–14.74)	-	
OR_a_ ^3^ (95% IC)	1	0.89 (0.05–14.87)	-	

OR_c_ = crude odds ratio. ^1^ Missing information from 1 individual. ^2^ *p*-value obtained from the χ^2^ test (Pearson *p*-value) or Fisher’s exact test, when needed. ^3^ Odds ratio adjusted (ORa) for age and Hg exposure levels. ^4^ Brief cognitive screening battery.

## Data Availability

Not applicable.
